# Transtibial Medial Meniscus Root Repair With Simplified Cross‐Bar Suture Configuration and Knotless Centralization

**DOI:** 10.1002/atn2.70324

**Published:** 2026-10-12

**Authors:** Charles Qin, Neal Goldenberg, Gregory Leather

**Affiliations:** ^1^ Department of Orthopedic Surgery Cheshire Medical Center Dartmouth Hitchcock Keene New Hampshire U.S.A.

## Abstract

The significance of medial meniscus root tears on articular cartilage degeneration of the medial compartment is well recognized. There is a consensus among peer‐reviewed literature on the importance of tear fixation over partial meniscectomy or observation in the appropriately indicated patient. Recent attention has been paid to the importance of reduction of meniscus extrusion through centralization techniques, as well as the effect of a lax repair on osteoarthritic progression and clinical outcomes. In this technical note and video, we describe our technique on transtibial medial meniscus root repair via a simplified cross‐bar configuration and knotless centralization of the meniscus.

## Graphical abstract

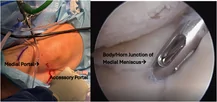

**VIDEO 1:** This case involves a 67‐year‐old active female patient who presents with acute medial and posterior knee pain after feeling a pop on rising from a crouched position. On examination, she had a knee effusion with hyperflexion pain. Weight‐bearing radiographs showed Kellgren‐Lawrence grade 2 changes in the medial compartment with less than 3° of varus malalignment. Magnetic resonance imaging showed a full‐thickness medial meniscus root tear with 4 mm of extrusion. On induction of general anesthesia, the patient is positioned supine with a lateral post. A post for the foot is also used to control knee flexion for final tensioning of the root repair. A standard 2‐portal diagnostic arthroscopy reveals a full‐thickness medial meniscus root tear with extrusion. The tear is prepared with a rasp to assist with the biological capacity to heal. We start with centralizing the meniscus at the body/horn junction first. The camera is moved to the medial portal, and a 15° labral elevator (Arthrex, Naples, FL) is inserted through the lateral portal which provides in‐line access to liberate the meniscotibial ligament and allow for the necessary mobility to correct extrusion. A spinal needle is used to plan the location of the accessory portal and the angle of insertion of the centralization anchor. Usually, this is 1 cm superior and 2 cm posterior to the medial portal. If the desired position is not immediately identified, multiple passes with the spinal needle on the medial side of the knee will create the unintended benefit of medial collateral ligament lengthening and improved access to the posteromedial aspect of the knee. The guide for the 1.8‐mm knotless Q‐FIX anchor (Smith & Nephew, Andover, MA) is inserted through the small accessory medial portal. The metal guide allows for superior leverage of the drill to avoid skiving when drilling for the anchor. The 1.8‐mm anchor is seated with radial expansion of the suture. At this point, the lateral portal is enlarged to accommodate a 7‐mm cannula. Because the centralization is accomplished with a horizontal mattress suture configuration, we find that the cannula is critical to prevent soft tissue bridges with the multiple passes required to obtain the horizontal mattress. The repair suture of the knotless anchor (minitape) is retrieved from the lateral portal. First, the minitape is passed through the meniscus tissue at the body/horn junction from inferior to superior with a self‐retrieving antegrade suture passer (FirstPass Mini, Smith & Nephew). Next, a shuttling suture is used to shuttle the minitape from superior to inferior to create a horizontal mattress configuration. An alternative option would be to forgo the shuttling suture and instead pass the minitape with the FirstPass Mini upside down. The disadvantage of this option is that the needle of the suture passer may hit the tibial surface, potentially preventing successful firing. The looped relay suture of the knotless anchor is retrieved through the lateral portal, and the shuttling suture is left docked in the accessory portal to allow for more in‐line pulling of the shuttling suture to complete the centralization. The tail of the repair minitape is cut. With the centralization complete, attention is now turned toward the root repair. Via the FirstPass Mini, a minitape is placed through the middle third zone of the root tissue in horizontal mattress configuration with the exact same technique as described earlier. A looped minitape is placed between horizontal mattress sutures in the peripheral zone of the root. This effectively creates a ripstop in a simplified cross‐bar configuration. A 55° offset guide (Smith & Nephew, Andover, MA) is used to drill a 2.8‐mm tunnel from the anterolateral surface of the tibia to the native root insertion, which had been prepared previously with a motorized shaver (Arthrex). The drill is cycled to remove any bony debris at the tunnel aperture that may affect future suture shuttling. The drill is removed, leaving a metal cannula in place through which a shuttling nitinol wire is delivered up into the joint. The wire is retrieved along with the minitapes through a cannula, and then, the minitapes are shuttled down the tunnel. The tapes are sequentially tensioned and loaded into a 3.5‐mm PEEK anchor (FootPrint, Smith & Nephew). The anchor is seated at 60° of knee flexion, completing the repair. Video content can be viewed at https://doi.org/10.1002/atn2.70324.

The significance of medial meniscus root tears on articular cartilage degeneration of the medial compartment is well recognized.^1^, ^2^ Laprade et al. have previously described a classification technique of these tears.^3^ There is a consensus among peer‐reviewed literature on the importance of tear fixation over partial meniscectomy or observation in the appropriately indicated patient.^4^ In summary, medial meniscus root repair is a cornerstone of joint preservation in the middle‐aged adult.

The effect of medial meniscus root repair on improving clinical outcomes and slowing down the progression of arthritic change is well documented. Recent attention has been paid to the importance of reduction of meniscus extrusion through centralization techniques, as well as the effect of a lax repair on osteoarthritic progression and clinical outcomes.^5^, ^6^ In our experience, we perform a centralization of the medial meniscus during root repair when preoperative meniscus extrusion is 3 mm or greater. Centralization is accomplished through a knotless suture anchor technique, which has been shown to be more effective in reducing extrusion than transtibial tunnel techniques.^7^ For the root repair itself, to attempt to achieve a repair resistant against loosening with cyclical displacement, we use a simplified cross‐bar suture configuration with low‐profile tape that involves placing a cinch suture across a horizontal mattress that effectively functions as a ripstop.

In this video, we present our technique on transtibial medial meniscus root repair via a simplified cross‐bar configuration and knotless centralization of the meniscus. We find that the low‐profile tape has the advantage of a broader footprint of the tape but is small enough to potentially reduce the risk of tissue strangulation particularly in the setting of degenerative root tissue.

This case involves a 67‐year‐old active female patient who presents with acute medial and posterior knee pain after feeling a pop on rising from a crouched position. On examination, she had a knee effusion with hyperflexion pain. Weight‐bearing radiographs showed Kellgren‐Lawrence grade 2 changes in the medial compartment with less than 3° of varus malalignment. Magnetic resonance imaging showed a full‐thickness medial meniscus root tear with 4 mm of extrusion.

## SURGICAL TECHNIQUE

On induction of general anesthesia, the patient is positioned supine with a lateral post. A post for the foot is also used to control knee flexion for final tensioning of the root repair.

A standard 2‐portal diagnostic arthroscopy reveals a full‐thickness medial meniscus root tear with extrusion (Video 1). The tear is prepared with a rasp to assist with the biological capacity to heal. We start with centralizing the meniscus at the body/horn junction first. The camera is moved to the medial portal, and a 15° labral elevator (Arthrex, Naples, FL) is inserted through the lateral portal which provides in‐line access to liberate the meniscotibial ligament and allow for the necessary mobility to correct extrusion. A spinal needle is used to plan the location of the accessory portal and the angle of insertion of the centralization anchor. Usually, this is 1 cm superior and 2 cm posterior to the medial portal (Figure 1). If the desired position is not immediately identified, multiple passes with the spinal needle on the medial side of the knee will create the unintended benefit of medial collateral ligament lengthening and improved access to the posteromedial aspect of the knee. The guide for the 1.8‐mm knotless Q‐FIX anchor (Smith & Nephew, Andover, MA) is inserted through the small accessory medial portal. The metal guide allows for superior leverage of the drill to avoid skiving when drilling for the anchor. The 1.8‐mm anchor is seated with radial expansion of the suture. At this point, the lateral portal is enlarged to accommodate a 7‐mm cannula. Because the centralization is accomplished with a horizontal mattress suture configuration, we find that the cannula is critical to prevent soft tissue bridges with the multiple passes required to obtain the horizontal mattress. The repair suture of the knotless anchor (minitape) is retrieved from the lateral portal. First, the minitape is passed through the meniscus tissue at the body/horn junction from inferior to superior with a self‐retrieving antegrade suture passer (FirstPass Mini, Smith & Nephew). Next, a shuttling suture is used to shuttle the minitape from superior to inferior to create a horizontal mattress configuration. An alternative option would be to forgo the shuttling suture and instead pass the minitape with the FirstPass Mini upside down. The disadvantage of this option is that the needle of the suture passer may hit the tibial surface, potentially preventing successful firing. The looped relay suture of the knotless anchor is retrieved through the lateral portal, and the shuttling suture is left docked in the accessory portal to allow for more in‐line pulling of the shuttling suture to complete the centralization (Figure 2). The tail of the repair minitape is cut.

**FIGURE 1:**
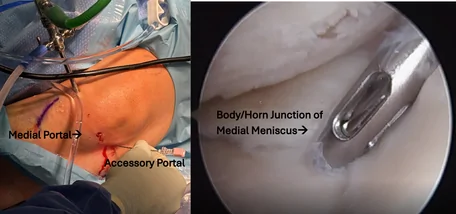
The arthroscope is placed in the lateral portal of the right knee for viewing. The body/horn junction of the medial meniscus is identified. An accessory medial portal (roughly 2 cm posterior and 1 cm superior) is localized via a spinal needle. Through this portal, the drill guide for the knotless anchor is inserted and placed onto the tibial surface just adjacent to the body horn junction.

**FIGURE 2:**
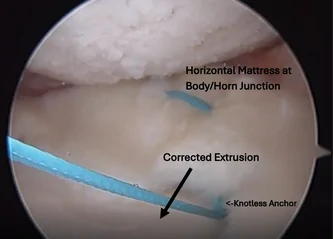
The arthroscope is in the medial portal of the right knee. At this point, the repair suture tape from the knotless anchor has been passed through the medial meniscus at the body horn junction in a horizontal mattress configuration. The repair suture tape has been shuttled back into the anchor completing the knotless construct. By tensioning the suture tape, the meniscus is centralized toward the anchor, correcting the extrusion.

With the centralization complete, attention is now turned toward the root repair. Via the FirstPass Mini, a minitape is placed through the middle third zone of the root tissue in horizontal mattress configuration with the exact same technique as described earlier. A looped minitape is placed between horizontal mattress sutures in the peripheral zone of the root. This effectively creates a ripstop in a simplified cross‐bar configuration (Figure 3). A 55° offset guide (Smith & Nephew) is used to drill a 2.8‐mm tunnel from the anterolateral surface of the tibia to the native root insertion, which had been prepared previously with a motorized shaver (Arthrex). The drill is cycled to remove any bony debris at the tunnel aperture that may affect future suture shuttling. The drill is removed, leaving a metal cannula in place through which a shuttling nitinol wire is delivered up into the joint. The wire is retrieved along with the minitapes through a cannula, and then, the minitapes are shuttled down the tunnel. The tapes are sequentially tensioned and loaded into a 3.5‐mm PEEK anchor (FootPrint, Smith & Nephew). The anchor is seated at 60° of knee flexion, completing the repair. A list of advantages and disadvantages to this technique is shown in Table 1.

**FIGURE 3:**
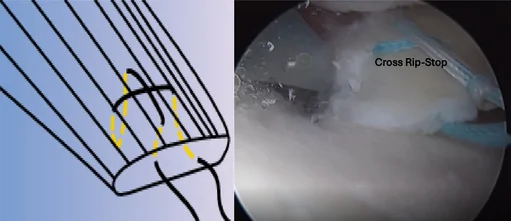
The arthroscope is in the medial portal of the right knee. An offset drill guide has been inserted at the native root insertion. The suture configuration for the root repair is visualized just medial to the drill guide. This is a simplified cross ripstop with a horizontal mattress followed by a luggage tag cinch suture. This suture configuration is shown in the drawing on the left.

**TABLE 1:** Advantages and Disadvantages

**Advantages**	**Disadvantages**
MCL lengthening can assist with visualization.	Poor visualization of the posterior aspect of the medial meniscus can make suture passage for both centralization and repair challenging with this technique.
Placement of the camera through the medial portal and the use of the lateral portal as the working portal provide in‐line access to the body/horn junction of the meniscus during centralization.	Failure to have in‐line direct access to the meniscus for centralization may lead to ineffective correction of meniscal extrusion.
Use of a cannula during suture placement in horizontal mattress configuration minimizes the risk of soft tissue bridges.	The horizontal mattress suture requires the repair suture to be passed twice with or without a shuttling suture, increasing the risk of a soft tissue bridge to occur.

MCL, medial collateral ligament.

## DISCUSSION

A successful outcome after medial meniscus root repair is driven by multiple factors. Careful evaluation of weight‐bearing radiographs, chondral status in the medial compartment on preoperative magnetic resonance imaging, assessment of body mass index, and limb alignment are critically important for patient selection.^8^ Proper rehabilitation compliance is equally important. With regard to surgical technique, we believe that the current evidence has identified repair laxity and persistent extrusion as key factors for suboptimal postoperative outcomes.^5^, ^9^

Variants of a ripstop suture configuration have been described in medial meniscus root repair in an effort to prevent suture loosening over time and allow for proper root healing. In our technique, the horizontal mattress ripstop suture serves to reinforce the vertical cinch suture repair, reducing the likelihood of suture pull‐out as well as repair loosening. The “tie‐grip” suture in radial repairs, similarly combining vertical and horizontal fixation techniques, has been shown to be most resistant to gapping.^10^

There are multiple described techniques of centralization, including transtibial and staple techniques.^11^ Although some evidence supports the use of knotless centralization over other techniques, we believe that regardless of technique chosen, there are a few critical factors that allow for successful centralization to correct extrusion. First, in‐line access to the meniscus with a labral elevator allows for liberation of the scarred meniscotibial ligament to achieve the necessary mobility to allow for centralization. Second, placement of the inverted horizontal mattress suture through the meniscus should be properly spaced to avoid bunching as well as stress concentration through 1 region of the meniscus. Finally, adding additional anchors may be needed in severe cases of extrusion involving the body and anterior horn.

In summary, we have developed our technique for transtibial medial meniscus root repair to avoid repair loosening and persistent meniscus extrusion. Further investigation is warranted to compare short‐ and long‐term outcomes across repair techniques.

## DISCLOSURES

The authors (C.Q., N.G., G.L.) declare that they have no known competing financial interests or personal relationships that could have appeared to influence the work reported in this article.
